# Macrolide-Resistant *Shigella sonnei*

**DOI:** 10.3201/eid1408.080147

**Published:** 2008-08

**Authors:** Leyla Boumghar-Bourtchai, Patricia Mariani-Kurkdjian, Edouard Bingen, Ingrid Filliol, Anne Dhalluin, Shadia Ait Ifrane, François-Xavier Weill, Roland Leclercq

**Affiliations:** * Centre Hospitalier Universitaire Côte de Nacre, Caen, France; † Hôpital Robert Debré, Paris, France; ‡Institut Pasteur, Paris; § Université de Caen, Caen, France

**Keywords:** Antibiotic resistance, shigellosis, surveillance, outbreak, azithromycin, dispatch

## Abstract

*Shigella sonnei* UCN59, isolated during an outbreak of *S. sonnei* in January 2007, was resistant to azithromycin (MIC 64 mg/L). The isolate contained a plasmid-borne *mph*(A) gene encoding a macrolide 2′-phosphotransferase that inactivates macrolides. Emergence of the *mph*(A) gene in *S. sonnei* may limit usefulness of azithromycin for treatment of shigellosis.

Shigellosis remains a common gastrointestinal disease in developing and industrialized countries. It occurs mostly in children <5 years of age; *Shigella sonnei* is the most frequently isolated species (*1*). Ampicillin and trimethoprim-sulfamethoxazole alleviate the dysenteric syndrome of shigellosis and reduce the infectious period. However, current resistance patterns limit the use of these drugs (*2*). Although fluoroquinolones are an effective alternative for adults, they are not approved for shigellosis treatment in children <18 years of age because of their potential toxicity (*2*,*3*). Azithromycin, a macrolide, represents an attractive treatment option for several reasons. It has in vitro activity against most *Shigella* spp. isolates (*4*), can be given once a day, and attains high intracellular concentrations (*5*). Despite MICs from 2 to 8 mg/L for *Shigella* spp., sufficient concentrations of azithromycin in the colon may inhibit *Shigella* spp. growth (*6*). Azithromycin is recommended by the American Academy of Pediatrics for treatment of shigellosis in children, by the World Health Organization as a second-line treatment in adults, and, since June 2004, by the Agence Française de Sécurité Sanitaire des Produits de Santé (*2*,*7*; www.agmed.sante.gouv.fr/htm/10/filcoprs/mp040601.pdf). In 1996, 2002, 2003, and 2007, outbreaks of shigellosis caused by *S. sonnei* resistant to ampicillin and trimethoprim-sulfamethoxazole occurred in children in northern Paris. The outbreaks occurred in religious schools, similar to cyclic outbreaks in US Jewish schools related to secondary transmission (*8*,*9*).We report an outbreak of shigellosis in and around Paris, France, in which azithromycin failure was related to emergence of plasmid-mediated resistance to macrolides.

## The Study

On January 24, 2007, *S. sonnei* strain UCN59 was isolated from a 4-year-old girl admitted to Robert Debré Hospital, Paris, for bloody diarrhea and fever. The strain was resistant to ampicillin, trimethoprim, sulfonamides, and cotrimoxazole but susceptible to quinolones, third-generation cephalosporins, and doxycycline according to the disk-diffusion technique. MICs of macrolides were markedly increased for *S. sonnei* UCN59 compared with those for a susceptible control *S. sonnei* UCN62 (Table). From January to April 23, 2007, a total of 50 cases of laboratory-confirmed shigellosis were identified. Isolates included, in addition to UCN59, 31 *S. sonnei* that had an azithromycin MIC >64 mg/L from 31 children <15 years of age, who had each been prescribed azithromycin for diarrhea. All patients lived in the Paris area and attended 8 religious schools.

**Table:** Macrolide susceptibility of outbreak and control *Shigella* isolates and *Escherichia coli* constructs

Strain	MIC, mg/L			
	Erythromycin	Clarithromycin	Azithromycin	Telithromycin
*Shigella sonnei* UCN 62	64	32	2	8
*S. sonnei* UCN 59	1,024	1,024	64	64
*Escherichia coli* K12 AG100A	2	2	2	1
*E. coli* K12 AG100A/pUV21	512	512	8	32
*E. coli* K12 AG100A/pUC18Ω*mph*(A)	>1,024	512	128	512

**Figure 1:**
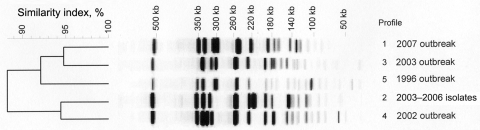
Pulsed-field gel electrophoresis–generated dendrogram for 43 *Shigella sonnei* isolates obtained from sporadic or outbreak cases during 1996–2007 in the Paris area. Profile 1) representative isolates from the 2007 outbreak, including 32 isolates with azithromycin MIC >256 mg/L by Etest and 2 isolates with azithromycin MIC <16 mg/L. Profile 2) 6 representative isolates from sporadic cases (2003–2006) with azithromycin MIC <16 mg/L. Profile 3) representative isolate Shi 03-3580 from 2003 outbreak with azithromycin MIC <16 mg/L by Etest. Profile 4) representative isolate Shi 02-9633 from 2002 outbreak with azithromycin MIC <16 mg/L by Etest. Profile 5) representative isolate Shi 96 1420 from 1996 outbreak with azithromycin MIC <16 mg/L by Etest.

**Figure 2:**
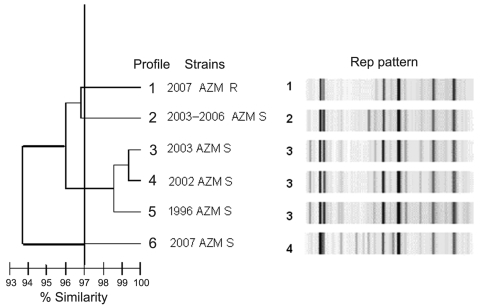
Repetitive sequence–based, PCR–generated dendrogram for 43 *Shigella sonnei* isolates obtained from sporadic or outbreak cases during 1996–2007 in the Paris area. Isolates with >97% similarity were considered to be closely genetically related. Profile 1) representative of the 32 isolates of the 2007outbreak with azithromycin (MIC >256 mg/L by Etest) . Profile 2) 1 of 6 isolates from sporadic cases (2003–2006) with azithromycin MIC <16 mg/L. Profile 3) representative isolate Shi 03-3580 from 2003 outbreak with azithromycin MIC <16 mg/L by Etest. Profile 4) representative isolate Shi 02-9633 from 2002 outbreak with azithromycin MIC <16 mg/L by Etest. Profile 5) representative isolate Shi 96 1420 from 1996 outbreak with azithromycin MIC <16 mg/L by Etest. Profile 6) isolate from 2007 with azithromycin MIC <16 mg/L by Etest; another AZM S 2007 isolate had an identical profile. AZM, azithromycin; R, resistant; S, sensitive; Rep, repetitive sequence-based PCR.

Typing by pulsed-field gel electrophoresis (PFGE) and repetitive sequence–based PCR (rep-PCR) by using the automated DiversiLab system (bioMérieux, La-Balme-les-Grottes, France) (*10*) was performed on the 32 azithromycin-resistant and on 11 azithromycin-susceptible (MIC <16 mg/L) sporadic or outbreak isolates obtained during 1996–2007 in the Paris area. Five different PFGE patterns were obtained by using the enzyme *Bln*I. All the 2007 outbreak isolates, including the 32 azithromycin-resistant isolates and 2 azithromycin-susceptible isolates, were clustered into a single profile, profile 1 (Figure 1). The presence of azithromycin-susceptible isolates with profile 1 was detected among the 1996 and 2002–2006 outbreak isolates (data not shown), showing the persistence of this clonal type over 10 years in this area of Paris. Other isolates displayed PFGE types 2 to 5. Low diversity of PFGE profiles was consistent with isolation of strains in the same area and for most of them from the same community. Four different patterns with <97% similarity were distinguished by rep-PCR (Figure 2). Again, all the 2007 azithromycin-resistant isolates were clustered; however, they could be distinguished from the 2007 azithromycin-susceptible isolates. In contrast to PFGE findings, rep-PCR showed that isolates representative of the 1996, 2002, and 2003 outbreaks were genetically related.

The *mph*(A) gene, which encodes a macrolide 2′-phosphotransferase that inactivates macrolide antimicrobial drugs, was amplified from *S. sonnei* UCN59 DNA by PCR (*11*). PCR was negative for the *erm*(A), *erm*(TR), *erm*(B), *erm*(C), and *erm*(X) methylase genes; the *ere*(A), *ere*(B) genes encoding esterases; the *mph*(B) gene encoding a phosphotransferase; and the efflux genes *mef*(A) and *msr*(A). Sequence of the genes that encode ribosomal structures composing the target of macrolides, *rrl*, *rplD,* and *rplV* genes in *S. sonnei* UCN59, did not display any mutation in the critical bases of resistance to macrolides.

The genes conferring resistance to ampicillin and erythromycin were transferred en bloc by conjugation from *S. sonnei* UCN59 to a macrolide-susceptible mutant *Escherichia coli* AG100A at a frequency of ≈10^–3^ per donor cell-forming unit after the mating period. A single plasmid was extracted from a transconjugant *E. coli* AG100A/pUV21. After restriction analysis, its size was estimated at ≈90 kb. PCR experiments showed that this plasmid belonged to incompatibility group I (*12*). MICs of macrolides for *E. coli* AG100A/pUV21 confirmed that this plasmid conferred cross-resistance to macrolides (Table).

*Eco*RI-restricted fragments of plasmid pUV21 were transferred to a nylon membrane and hybridized to an *mph*(A) probe. The *mph*(A) gene was borne by an ≈20-kb *Eco*RI fragment, confirming that resistance to azithromycin was plasmid mediated.

After plasmid digestion with *Pst*I enzyme, a DNA fragment that conferred resistance to erythromycin was cloned in plasmid pUC18 and introduced by transformation into *E.* *coli* AG100A to generate *E. coli* K12 AG100A/pUC18Ω*mph*(A). Sequence of the inserted DNA was determined. The fragment contained 4 open reading frames (ORFs) in the same orientation: *mph*(A), *mrx* that putatively encodes a membrane protein, *mphR*(A) that regulates the expression of *mph*(A), and an ORF of unknown function. This series of ORFs was flanked by a copy of IS*26* at the 5′ end and a copy of IS*6100* at the 3′ end. BLAST analysis (www.ncbi.nlm.nih.gov/blast/Blast.cgi) showed that the nucleotide sequence was nearly identical to that of fragments of plasmid pU302L from *Salmonella* *enterica* serotype Typhimurium (C.Y. Chen et al., unpub. data, GenBank accession no. NC_006816), of *Shigella flexneri* transposon TnSF1 (J.H. Chen and J.Y. Chen, unpub. data, GenBank accession no. AF188331), and of plasmids pRSB101 and pSRB107 (*13*,*14*).

## Conclusions

Few data are available on azithromycin resistance in *Shigella* spp. A recent report from Bangladesh mentioned that 16% of *Shigella* isolates were resistant to azithromycin and that 62% had intermediate resistance according to the Clinical Laboratory Standards Institute breakpoints recommended for streptococci (>1 mg/L, resistant; <0.25 mg/L, susceptible) (*15*). However, the MIC_90_ (MIC at which 90% are susceptible) of 8 mg/L displayed by the microorganisms was within the normal range of MICs for this microorganism; no isolate had an azithromycin MIC >24 mg/L, which suggests that none had acquired resistance to azithromycin. Surveillance for resistance to azithromycin in *Shigella* spp. requires specific breakpoints for this species (*3*).

The *mph*(A) gene has been detected in the sequence of transposon TnSF1 isolated from *S. flexneri* (J.H. Chen and J.Y. Chen JY, unpub. data, GenBank accession no. AF188331). The *mph*(A) gene was first reported in an *E. coli* isolate from Japan (*10*). Since then, the gene has been found in *Aeromonas hydrophila*, *Pseudomonas* spp., *Stenotrophomonas* spp., and a variety of enterobacteria (listed at http://faculty.washington.edu/marilynr/ermweb4.pdf).

Azithromycin was used to treat shigellosis in France only after the release of the French recommendations in 2004. Subsequent rapid emergence of azithromycin-resistant isolates may be a limitation for the use of macrolides in shigellosis. Because use of azithromycin is proposed for treatment of shigellosis, susceptibility of the isolates to azithromycin should be routinely tested.
